# Young adult donor bone marrow infusions into female mice postpone age-related reproductive failure and improve offspring survival

**DOI:** 10.18632/aging.100002

**Published:** 2008-11-14

**Authors:** Kaisa Selesniemi, Ho-Joon Lee, Teruko Niikura, Jonathan L. Tilly

**Affiliations:** Vincent Center for Reproductive Biology, Vincent Obstetrics and Gynecology Service, Massachusetts General Hospital/Harvard Medical School, Boston, MA 02114, USA

**Keywords:** stem cell, bone marrow, ovary, fertility, aging, reproduction, menopause

## Abstract

The female reproductive axis is the first major organ system of the body to fail with advancing age. In addition to a permanent cessation of fertile potential, the loss of cyclic ovarian function in humans heralds the onset of menopause, which in turn underlies the emergence of a diverse spectrum of health issues in aging women. Recently, it was reported that bone marrow (BM) transplantation (BMT) into adult female mice conditioned a week earlier with highly cytotoxic drugs rescues ovarian function and fertility. Herein we show in mice receiving no prior conditioning regimen that once-monthly infusions of BM-derived cells retrieved from young adult female donors bearing an enhanced green fluorescent protein (EGFP) transgene sustain the fertile potential of aging wild-type females long past their time of normal reproductive senescence. The fertility-promoting effects of female donor BM are observed regardless whether the infusions are initiated in young adult or middle-aged females. Although the mechanism by which BM infusions benefit the reproductive performance of aging females remains to be elucidated, the absence of EGFP-expressing offspring suggests that it does not depend on development of mature eggs derived from germline-committed cells in the donor marrow. However, donor BM-derived somatic cells accumulate in the recipients, indicating efficient donor cell engraftment without prior conditioning. These findings provide a strong impetus to further explore development of adult stem cell-based technologies to safely extend function of the female reproductive axis into advanced age without the need for toxic pre-conditioning protocols routinely used in other models of stem cell delivery.

## Introduction

Declining health in aging individuals reflects both the impaired function of a given organ, which in turn yields a disorder specific to that organ, as well as the breakdown of inter-organ communication networks controlled primarily by hormonal signals. The ovaries represent a classic example of both situations in that the female gonads serve as the source of not only germ cells (oocytes) needed for reproduction, but also a large number of bioactive factors that support or modulate the function of many other tissues and cells [1,2].

Unfortunately, the ovaries are the first major organs to fail in aging females, and this occurs long before age-related dysfunction of other tissues is observed. For example, in women fertility becomes severely compromised around the age of forty [3], preceding the menopause by about a decade. Female mice exhibit a similar impairment of fertile potential approximately halfway through their chronological lifespan [4,5]. Irrespective of the species evaluated, ovarian failure and the ensuing loss of fertility are driven by depletion and ultimate exhaustion of the oocyte-containing follicle reserve [6].

Perhaps even more important than the loss of fertility, age-related ovarian failure sets the stage in aging females for markedly increased risks of developing a large number of debilitating health issues, including osteoporosis, cardiovascular disease and cognitive dysfunction [1]. In fact, recent studies in mice have solidified the direct causal association between ovarian failure and deteriorating health in females as they age. For example, inactivation of the pro-apoptotic Bax gene, which sustains the follicle pool and thus functional ovarian lifespan into very advanced age [7], extends fertile potential in aging females and minimizes the appearance of many age-related health problems, including bone and muscle loss, excess fat deposition, alopecia, cataracts, deafness, increased anxiety, and selective attention deficit [2]. Other studies have demonstrated that overall lifespan can be increased by transplanting young adult ovaries into aging female mice [8].

Despite the compelling nature of these findings, the fact that similar approaches are not feasible in humans has kept any possible clinical translation of this work uncertain. This may be on the verge of change, however, as new data suggest that ovarian function and fertility can be dramatically altered by technologies that might prove amenable for potential clinical development. The first of these data sets revolves around the surprising finding that the oocyte-containing follicle pool set forth at birth is, contrary to longstanding belief [9], replenished during adulthood by an as-yet unidentified population of presumptive female germline stem cells [10-13]. These findings have opened the possibility of developing new pharmacologic tools aimed at stimulating these cells to enhance oocyte formation when it might be clinically desirable, such as in females on the verge of reproductive failure [13,14]. Other studies with mice have shown that approaches known to repress the insulin/insulin-like growth factor signaling pathway, such as moderate dietary caloric restriction (CR) initiated in adulthood [5] or chronic treatment with the anti-diabetic compound metformin [15], can dramatically extend cyclic ovarian function and fertility into very advanced ages.

Regenerative medicine has also recently come into play in the context of female reproductive biology based on results showing that bone marrow (BM) transplantation (BMT) into chemotherapy-conditioned female mice generates a small number of donor-derived oocytes contained within immature follicles [11,16] but does not yield mature (fertilization competent) donor-derived eggs [16,17]. Similarly, spermatogonia, but not mature sperm, have been derived from BM of male mice [18,19] and men [20]. While these latter findings suggest that gametes arising from BM-derived cells exhibit a maturational defect, clinical studies have linked BMT to a return of gonadal function and fertility in some cancer survivors following high-dose chemotherapy [21-27]. These data and results from a recent study reporting a comparable rescue of long term-fertility in chemotherapy-treated female mice following BMT [16] further support that adult stem cell-based technologies may provide a novel means to restore or sustain reproductive organ function. Herein we tested in mice if once-monthly intravenous BM infusions (BM-INF), administered without prior radiation or chemotherapy conditioning, could delay age-related failure of the female reproductive axis.

## Results

### Repeated BM-INF sustain natural fertility of aging

In initial experiments to examine the impact of BM-INF on function of the female reproductive axis with age, BM was harvested from young adult (6-10 weeks of age) female C57BL/6 donors and intravenously infused into non-conditioned female C57BL/6 recipients once every 4 weeks (n = 20). Thirteen of the recipients received BM collected from wild-type females, whereas the remaining 7 recipients received BM harvested from young adult transgenic donor females expressing enhanced green fluorescent protein (EGFP) under the control of a non-cell lineage-specific promoter [β -actin-EGFP; JAX strain C57BL/6-Tg(ACTB-EGFP)1Osb/J]. Vehicle infusions (VEH-INF) into age-matched C57BL/6 females (n = 20) were performed in parallel. The infusions were initiated at 3 months of age, as past studies have shown that a significant decline in the primordial follicle pool in adult C57BL/6 female mice does not occur until after 100 days of age [12]. Hence, this design minimized the chance of a significant depletion of the follicle reserve in adulthood prior to initiation of the infusions. Furthermore, all females were mated to assure their fertility prior to initiation of the first infusions (data not shown).

**Figure 1.**
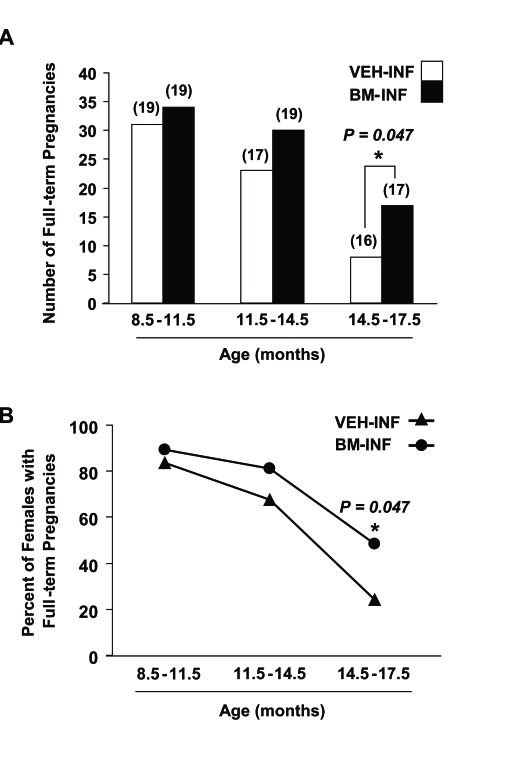
Repeated BM-INF delay female reproductive aging. **(A)** Number of full-term pregnancies achieved by female mice at the indicated ages following once-monthly infusions of vehicle (VEH-INF) or BM harvested from young adult female donors (BM-INF), initiated at 3 months of age. The total number of recipients analyzed in each age bracket is indicated in parentheses over the respective bars. **(B)** Percentage of VEH-INF and BM-INF females that achieved full-term pregnancies between 8.5-11.5, 11.5-14.5 and 14.5-17.5 months of age, as calculated from the raw data shown in panel A.

Between 3-8.5 months of age (prime reproductive life), VEH-INF (n = 20) and BM-INF (n = 20) females achieved a total of 56 and 59 full-term pregnancies, respectively, leading to the birth of offspring (data not shown). Between 8.5-11.5 months of age (transitional period leading up to reproductive failure), 19 remaining VEH-INF and 19 remaining BM-INF females delivered a total of 31 and 34 litters, respectively (Figure 1A). However, between 11.5-14 months of age (beginning of reproductive failure), 30 full-term pregnancies were achieved by the 19 remaining BM-INF females, whereas only 23 full-term pregnancies were achieved  by the 17 remaining VEH-INF females (Figure 1A). Importantly, the two additional females that died in the VEH-INF group between 11.5-14 months of age had their last pregnancies at an approximately 8 months of age (data not shown). Thus, even if these two females were still present, it would be highly unlikely that they would have increased the total number of full-term pregnancies achieved by the VEH-INF females during this age bracket. The fertility of VEH-INF females declined even further between 14.5-17.5 months of age, with only 8 full-term pregnancies achieved by 16 remaining females. In comparison, 17 remaining BM-INF females delivered more than twice the number of litters between 14.5-17.5 months of age when compared with outcomes from the age-matched VEH-INF females (Figure 1A). Since the number of animals in each treatment group varied slightly with age, these data were re-calculated as a percentage of the total number of VEH-INF or BM-INF females that achieved full-term pregnancies during the indicated age brackets. This analysis reaffirmed that repeated BM-INF improved the reproductive performance of aging females, particularly between 14.5-17.5 months of age (Figure 1B).

### Fertility is benefited irrespective of when the BM-INF are initiated

**Figure 2.**
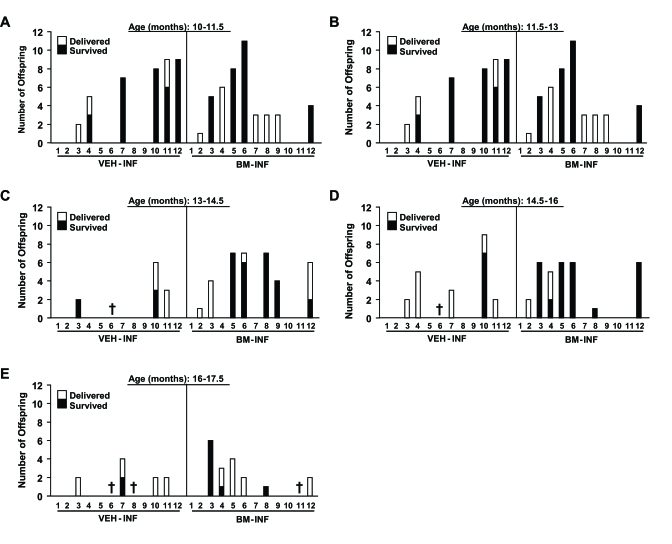
Reproductive performance of aging female mice after once-monthly infusions of vehicle or BM harvested from young adult female donors, initiated at 8 months of age. Fertility outcomes are shown for each VEH-INF female and BM-INF female between 10-11.5 **(A)**, 11.5-13 **(B)**, 13-14.5 **(C)**, 14.5-16 **(D)**, and 16-17.5 **(E)** months (M) of age (each mouse is represented by a number on the x-axis) run in parallel mating trials. The total number of offspring delivered and that survived for each female are indicated. Crosses designate mice that had to be euthanized due to severe health complications or that died of natural causes during the study period.

**Figure 3.**
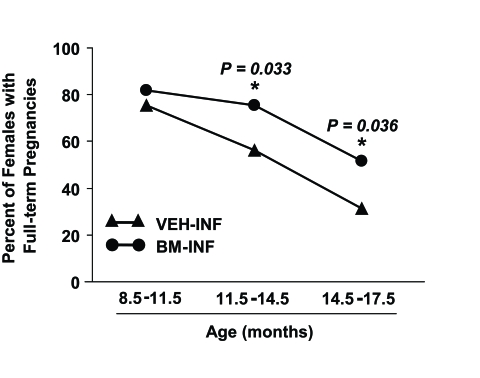
Pooled analysis of the effects of BM-INF on reproductive function in aging females. Percentage of VEH-INF and BM-INF females that achieved full-term pregnancies at the indicated ages, as calculated from the combined raw data shown in figure 1A (infusions started at 3 months of age) and figure 2 (infusions started at 8 months of age). A significantly (Fisher's exact test) higher percentage of BM-INF females achieved full-term pregnancies between 11.5-14.5 and 14.5-17.5 months of age compared with age-matched VEH-INF females.

The finding that repeated BM-INF sustained function of the female reproductive axis when the infusions were initiated early in adulthood (Figure 1) prompted us to examine whether fertility with age could be maintained if the infusions were initiated in females at the transitional period just prior to the onset of natural infertility. To test this, BM harvested from young adult β-actin-EGFP transgenic females or vehicle was infused into non-conditioned wild-type recipient females once every 4 weeks starting at 8 months of age, and mating trials were begun 2 months later. Between 10-11.5 months of age, similar numbers of VEH-INF and BM-INF females achieved full-term pregnancies (Figure 2A) However, for all subsequent mating attempts between 11.5-17.5 months of age, 2-4 additional mice in BM-INF group achieved full-term pregnancies when compared to the age-matched VEH-INF cohort (Figure 2B-E). Notably, and as will be discussed in more detail below, the number of offspring delivered by aging BM-INF females that survived postnatally was consistently higher than survival rates of offspring delivered by age-matched VEH-INF females (Figure 2B-E). Since a similar beneficial effect of BM-INF on reproductive capacity was observed irrespective of when in adult life the infusions were initiated, the results from the two trials were compiled and analyzed together as a percentage of females able to achieve full-term pregnancies in advanced ages (Figure 3). On average, 76% of 8.5-11.5-month-old, 56% of 11.5-14.5-month¬old, and 31% of 14.5-17.5 month-old VEH-INF females remained fertile (Figure 3). In comparison, 82% of 8.5-11.5-month-old, 75% of 11.5-14.5-month-old, and 52% of 14.5-17.5 month-old BM-INF females achieved full-term pregnancies and delivered offspring (Figure 3).

### Repeated BM-INF improve survival of offspring delivered by aged females   

**Figure 4.**
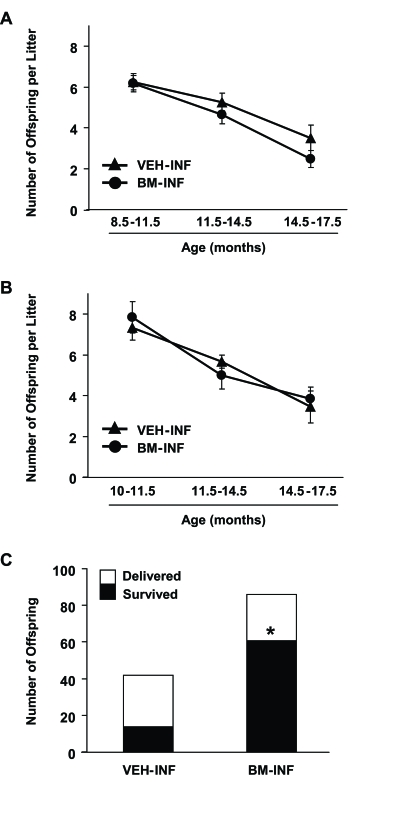
Repeated BM-INF do not affect fecundity but dramatically improve survival rates of offspring delivered by aging females. Summary of fecundity (mean ±SEM) of female mice that achieved full-term pregnancies at the indicated ages following once-monthly infusions of vehicle (VEH-INF) or BM harvested from young adult female donors (BM-INF), starting at 3 months **(A)** or 8 months **(B)** of age. **(C)** Offspring number and survival rates in mating trials of VEH-INF or BM-INF females between 10-17.5 months of age following once-monthly infusions initiated at 8 months of age. In addition to a marked increase in the total number of offspring delivered, the number of offspring delivered that survived was significantly increased by BM-INF (*, P = 0.0001 versus the VEH-INF group by Fisher's exact test).

During the entire mating period, females who received their first VEH or BM infusions at 3 months of age delivered a total of 765 and 849 offspring, respectively. Fecundity in both groups declined steadily with age from peak values of 7.8 ± 0.4 (VEH-INF) and 8.2 ± 0.3 (BM-INF) pups per litter at the beginning of the mating trials (data not shown) to 3.5 ± 0.6 (VEH-INF) and 2.5 ± 0.4 (BM-INF) pups per litter during the final mating trials (14.5-17.5 months of age) (Figure 4A). Similar outcomes were observed when the infusions were initiated at 8 months of age. A total of 126 and 185 offspring were delivered by VEH-INF and BM-INF females, respectively, between 10-17.5 months of age. Fecundity fell from 7.9 ± 0.8 (VEH-INF) and 7.3 ± 0.6 (BM-INF) pups  per litter at 10 months of  age to  3.4 ± 0.8 (VEH-INF) and 3.8 ± 0.6 (BM-INF) pups per litter between 14-17.5 months of age (Figure 4B). While no differences in fecundity were detected among the treatment groups, a striking difference in postnatal offspring survival was observed. Only 33% of the 42 offspring delivered by 13-17.5-month old VEH-INF females survived after delivery, whereas 71% of the 86 pups delivered by age-matched BM-INF survived (Figure 4C). Of final note, all offspring delivered by females infused with BM from β-actin-EGFP donors (n = 512) were genotyped and found to be derived from the recipient germline (data not shown).

### Chimerism analysis of the infused recipients

**Figure 5.**
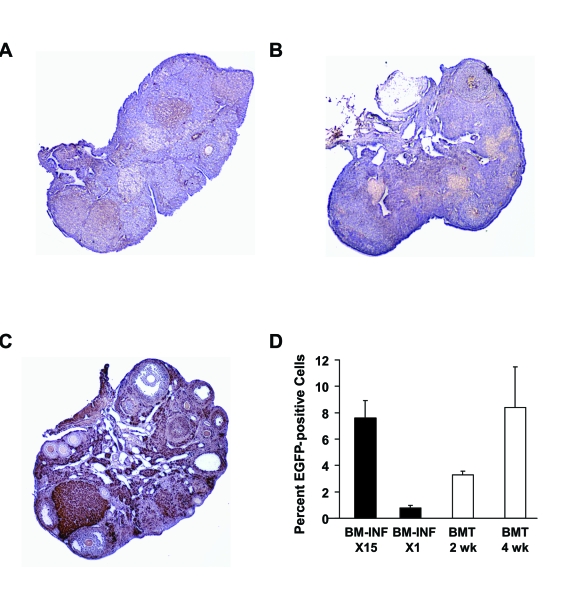
Analysis of donor cell engraftment in recipients after BM-INF without prior conditioning. Representative immunohistochemical analysis of EGFP expression (brown) in ovaries of aged wild-type females following 15 once-monthly infusions of BM harvested from young adult β-actin-EGFP transgenic female donors **(A, B)**. The ovary of a representative transgenic donor female **(C)** is shown as a positive control. **(D)** Chimerism analysis of BM-derived cells collected from female mice following 15 once-monthly infusions of β-actin -EGFP transgenic BM (BM-INF, X15) or a single infusion of β-actin-EGFP transgenic BM (BM-INF, X1). Parallel analysis of BM harvested from recipient females conditioned with busulfan and cyclophosphamide prior to BMT (again using β transgenic females as donors) is shown for comparison. For these samples, BM was collected 2 weeks (wk) and 4 weeks post-BMT. The data shown represent the mean ± SEM of results from analysis of 4 mice per group.

Donor cell tracking in the ovaries of females infused with BM from β-actin-EGFP donors was performed at the conclusion of the mating trials, and showed little evidence of EGFP-positive cell engraftment (Figure 5A-C).

Unfortunately, the advanced age of the females from which the ovaries were collected precluded in-depth analysis of germline chimerism, as has been reported for the ovaries of young adult chemotherapy-conditioned mice following stem cell transplantation [11,16]. Analysis of wild-type recipient BM by flow cytometry at the end of the mating trials (4 weeks after the last infusion) showed 7.6 ± 1.3% EGFP chimerism (n = 4, Figure 5D). The EGFP-positive cells found in the BM of once-monthly-infused female recipients at the end of the experiments (15 infusions total) were unlikely to represent only cells from the last infusion, as a single infusion of cells derived from BM of β-actin-EGFP donors into non-conditioned wild-type recipients resulted in 0.8 ± 0.2% chimerism 2-4 weeks later (n = 4; Figure 5D). For comparison, engraftment of EGFP-positive BM-derived cells was additionally analyzed in female recipient conditioned Under these conditions, 3.3 ± 0.2% (n = 4) and 8.4 ± 3.1% (n = 4) of the total recipient BM-derived cell population was found to be EGFP-positive at 2 and 4 weeks post-BMT, respectively (Figure 5D).

## Discussion

As human life-expectancy continues to trend upward, biomedical  research  directed  at  promoting improved health and well-being in aging individuals has become an increasingly relevant and widespread area of  scien tific investigation [28]. During the last few years, increasing interest has been placed on the possible use of regenerative medicine and adult stem cells, particularly those derived from BM, in combating age-related deterioration of organs and tissues. Much of our current understanding of adult stem cell biology in this regard, however, derives from studies in which stem or progenitor cells were tested for their ability to improve organ function in disease, injury, or insult models [16,29-33] or from in vitro studies in which BM-derived cells have been shown to differentiate into variety of cell types [33,34]. Thus, although a wealth of general information regarding the plasticity of BM-derived and other adult stem cells is available, very little is known of the potential utility of such cells to actually combat various aspects of physiological aging in vivo.

The female reproductive axis provides an excellent model for understanding age-related organ degeneration, as the ovaries undergo functional decline and failure relatively early in life, long before possible confounding influences from aging of the other tissues and organs [1,3]. Thus, in this study we used the female reproductive system as a model to investigate whether physiological decline in organ function with age could be postponed by in vivo delivery of cells harvested from young adult BM, which is known to be a rich source of stem cells. Our results show that once-monthly infusions of adult BM-derived cells into recipient female mice that received no prior cytotoxic conditioning regimen sustain the function of the female reproductive axis into advanced chronological age. Further, the improvements in reproductive performance with age were observed regardless of whether the infusions were initiated early in adult life (3 months of age) or in mid-adult life just prior to the decline in reproductive function (8 months of age). Interestingly, however, females receiving their first BM-INF at 8 months of age that were infertile from the beginning of mating attempts at 10 months of age were not able to regain their fertility. These findings indicate that BM-derived cells from young donors are effective at sustaining reproductive organ function only if administered at an age when the target tissue(s) is still functional.

**Figure 6.**
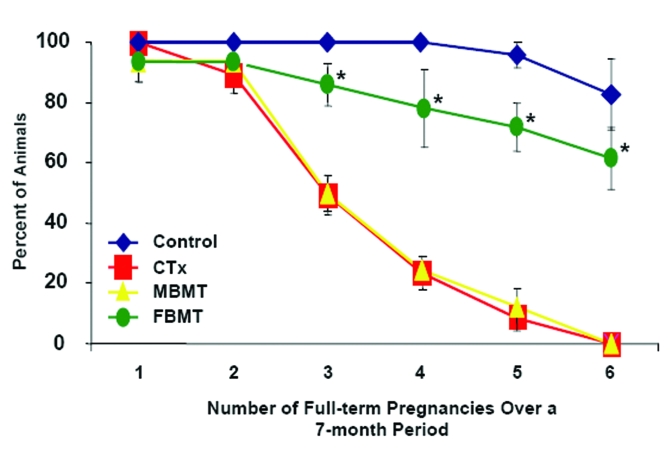
Male donor BM does not replicate the pro-fertility effects of female donor BM. Percentage of female mice receiving vehicle (Control, n = 18), a combination chemotherapy regimen containing busulfan and cyclophosphamide (CTx, n = 21) or CTx followed by bone marrow transplantation 1 week later, using young adult male (MBMT, n = 16) or female (FBMT, n = 19) donors, that achieved full-term pregnancies over a subsequent 7-month period when mating was initiated coincident with the transplants. Data shown are the mean ± SEM of combined results from 3 separate trials (*, P < 0.05 versus the respective CTx group or CTx + MBMT group).

The mechanisms by which the infusions of young adult BM cells into females postpone age-related infertility remain to be elucidated. While our previous studies with mice have shown that transplanted BM-derived cells can differentiate into immature oocytes and rescue long-term fertility of chemotherapy-treated recipient females [16], in this study we did not detect a significant level of donor cell chimerism (somatic or germline) in the ovaries of the recipients at the conclusion of the mating trials. Moreover, all the offspring delivered by females infused with BM from β-actin-EGFP-transgenic donors were wild-type and thus derived from the recipient germline. Although these data suggest that the infused BM-derived cells indirectly impact on the function of the female gonads with age, it is equally possible that EGFP chimerism in recipient ovaries was extremely low due to the advanced age of the tissue at the time of assessment. It has also recently been proposed from studies with rats that BM-derived cells secrete a variety of cytokines which improve ovarian function in recipient females after cytotoxic insult by, at least in part, reducing apoptosis in ovarian somatic cells [35]. If a similar situation exists in the non-insult model of aging, secreted factors from repeatedly infused young adult BM-derived cells could act as anti-apoptotic signals in the recipient ovaries or as stimulants to facilitate reactivation of stem cells in aging target tissues. While a case for the latter has recently been made from studies of improved muscle regeneration in aged mice after parabiotic blood exchange with younger but not aged animals [36], we have found in the chemotherapy model of induced ovarian failure [16] that BM harvested from male mice does not possess the same pro-fertility effects of transplanted female donor BM (Figure 6). Assuming there is no gender-specific difference in the ability of BM-derived cells to secrete various cytokines and other bioactive factors, these data suggest another mechanism underlies the fertility outcomes observed herein.

Whatever the case, the most striking, and to us unexpected, result of the repeated BM-INF was the dramatic improvement in the survival rates of pups delivered by aged females. Although fecundity (pups born per litter) was unaffected by BM-INF, the survival of offspring born to 13-17.5-month-old BM-INF recipients was far superior when compared to that of offspring delivered by age-matched VEH-INF females. To our knowledge, this is one of the first examples of an experimental approach conveying such an effect, other than moderate CR initiated in females during adulthood [5]. As is the case with the CR model, these results could be explained by one of several not mutually exclusive possibilities. The most logical is an improvement in the overall quality of eggs in aging females, since age-related deterioration in egg quality represents one of the most cumbersome issues faced by women of advanced maternal age trying to become pregnant [37,38]. It is also possible that non-ovarian target tissues of the aging female reproductive axis, such as the uterus where the embryo implants to form a placenta for fetal gestation, are benefited by repeated BM-INF. In support of this, recent studies of human females that received allogeneic BMT demonstrated the presence of donor-derived cells in the uterus of the recipients, albeit at low numbers [39,40]. Although more work is needed to fully understand the mechanisms at work, these findings support continued development of cell transplantation-based technologies to safely extend ovarian function and fertility of aging females. Furthermore, while much speculation exists regarding the potential for regenerative medicine to combat age-related organ failure, this study provides important proof-of-concept that such an outcome can actually be achieved and can be done without the need for toxic conditioning protocols used in other models of in-vivo BM or adult stem cell delivery.

## Methods

Animals. Wild-type 8-month-old C57BL/6 female mice were obtained from Taconic (Germantown, NY), whereas all young adult C57BL/6 female mice and C57BL/6 male mice were purchased from The Jackson Laboratories (Bar Harbor, ME). Breeding pairs of transgenic mice with EGFP expression driven by the chicken β-actin promoter were obtained from The Jackson Laboratories [JAX strain C57BL/6-Tg(ACTB-EGFP)1Osb/J]. All animal procedures reported herein were reviewed and approved by the institutional animal care and use committee of Massachusetts General Hospital.

Bone marrow infusions. Bone marrow was harvested from the femurs and tibias of female donor mice just prior to the infusions. Bones were cleaned and then crushed with a mortar and pestle in cold 1X-phosphate buffered saline (PBS). The cells were then passed through a 40-μm-filter, centrifuged and resuspended in ACK Lysing Buffer (Fisher Scientific, Pittsburgh, PA) for red blood cell lysis. The cells were centrifuged, washed and resuspended in cold PBS. Once isolated, 1.5-3X107 mononuclear cells (in 0.25 ml) were injected into recipients via the lateral tail vein as described [11,16], with the exception that the recipients were not conditioned with chemotherapy beforehand. Infusions were initiated at 3 or 8 months of age, and repeated every 4 weeks for the duration of the study. For single BM-INF, 1.5-3x107 cells derived from BM of β-actin-EGFP mice were infused into 8-week-old C57BL/6 (wild-type) female mice, and recipient BM was collected 2-4 weeks later for chimerism analysis.

Fertility testing (mating trials). All females whose infusions were initiated at 3 months of age were mated prior to the first infusion to assure fertility. For outcomes analysis, mating trials were initiated at 3 or 10 months of age under a paired breeding arrangement housing two females and one male of proven fertility in each cage. Males were randomly rotated among the cages during the mating trials [5]. The total number of offspring delivered per litter and the number of offspring delivered that survived were recorded separately for each pregnancy. Pups that did not survive were either found dead at birth or died very shortly after delivery. All viable offspring were allowed to remain with the dam until weaning, at which time the offspring were removed from the cages to allow for a subsequent mating attempt with the dam. For all offspring that survived, no anatomical or health complications were observed (data not shown).

Bone marrow transplantations . Eight-week-old C57BL/6 female mice were chemotherapy conditioned using 12 mg/kg busulfan (Sigma, St. Louis, MO) and 120 mg/kg cyclophospamide (Cytoxan; Bristol-Meyers Squibb, New York, NY), as described [11,16].  For cell trac-king experiments, 1 week post-cytotoxic drug treatment each female received a tail vein injection of vehicle or 1.5-3x107BM-derived cells from young adult β-actin-EGFP donor females. Recipients were euthanized at 2 and 4 weeks post-transplantation, and BM was har-vested and processed for flow cytometric analysis of EGFP chimerism. For fertility testing, 1 week post-cytotoxic drug treatment each female received a tail vein injection of vehicle or 1.5-3x107 BM-derived cells from young adult (8-10 weeks of age) wild-type male or female donors, and mating trials were conducted as described [16].

Donor cell tracking and EGFP chimerism in BM **.**For donor cell tracking, recipient ovaries were fixed in 4% paraformaldehyde, embedded in paraffin and sectioned for analysis using a GFP-specific antibody (sc-9996; Santa Cruz Biotechnology, Santa Cruz, CA), as detailed previously [11,16]. Positive and negative controls consisting of ovarian tissue from β-actin-EGFP transgenic females and wild-type C57BL/6 females, respectively, were analyzed in parallel on the same slides.

Bone marrow-derived cell preparations from the indicated experimental groups were prepared as detailed above for the BM infusions. After the last PBS wash, the cells were resuspended in cold PBS containing 1 µg/ml propidium iodide (Invitrogen, Carlsbad, CA) for exclusion of necrotic cells from the analysis. Live BM cells were analyzed for EGFP expression with a flow cytometer (FACSaria, BD Biosciences) at the Harvard Stem Cell Institute Flow Cytometry Core (Massachusetts General Hospital, Center for Regenerative Medicine, Boston, MA), as described previously [16]. As controls, BM samples from VEH-INF wild-type females, non-transplanted wild-type females and β-actin-EGFP transgenic females were analyzed in parallel (data not shown).

Data presentation and analysis . Graphs depict results from each individual mouse or combined data from the independent trials. Where appropriate, combined data were analyzed by Fisher's exact test (GraphPad Prism software, version 4.0; San Diego, CA) for statistical comparisons of results between experimental groups.
